# Study of the Fracture Behavior of Tetragonal Zirconia Polycrystal with a Modified Phase Field Model

**DOI:** 10.3390/ma13194430

**Published:** 2020-10-05

**Authors:** Jingming Zhu, Jun Luo, Yuanzun Sun

**Affiliations:** 1 Department of Mechanics, Huazhong University of Science and Technology, Wuhan 430074, China; jimizhu@hust.edu.cn (J.Z.); sun_yuanzun@163.com (Y.S.); 2 Hubei Key Laboratory for Engineering Structural Analysis and Safety Assessment, Luoyu Road, Wuhan 430074, China

**Keywords:** phase field method, crack propagation, transformation toughening, martensitic transformation, tetragonal zirconia polycrystal

## Abstract

The superior fracture toughness of zirconia is closely correlated with stress-induced martensitic phase transformation around a crack tip. In this study, a modified phase field (PF) model coupling phase transformation and fracture is proposed to study the fracture behavior and toughening effect of tetragonal zirconia polycrystal (TZP). The stress-induced tetragonal to monoclinic (t–m) phase transformation around a static or propagating crack is characterized with PF simulations. It is shown that the finite size and shape of the transformation zone under different loads and ambient temperatures can be well predicted with the proposed PF model. The phase transformation may decrease the stress level around the crack tip, which implies the toughening effect. After that, crack propagation in TZP is studied. As the stress field is perturbed by the phase transformation patterns, the crack may experience deflection and branching in the propagation process. It is found that the toughness of the grain boundaries (GBs) has important influences on the crack propagation mode. For TZP with strong GBs, the crack is more likely to propagate transgranularly while, for TZP with weak GBs, intergranular crack propagation is prevalent. Besides that, the crystal orientation and the external load can also influence the topology of crack propagation.

## 1. Introduction

Zirconia ceramics possess many superior mechanical properties such as high strength and toughness, excellent wear resistance, etc. The excellent mechanical properties of zirconia ceramics originate from the stress-induced tetragonal to monoclinic (t–m) martensitic transformation in the high-stress concentration zone around a crack tip. The unique transformation toughening behavior of zirconia was first discovered by Garvie et al. [1]. Since then, numerous experimental works were performed to reveal the toughening mechanisms in zirconia based ceramics [2,3,4,5,6,7,8,9,10,11]. In the meanwhile, quantitative models were set up to characterize the toughening effect of phase transformation in zirconia [12,13,14,15,16,17,18,19,20,21,22]. Several excellent reviews on this topic can be found in References [23,24,25,26,27]. It is now well recognized that both the dilatational and shear components of the transformation strain contribute to the toughening effect [12,13,28,29]. Most of the quantitative theoretical models are based on certain stress-based transformation criterion or macroscopic constitutive relations to predict geometric features of the transformation zone. The change of the stress intensity factor or the extra energy consumption due to phase transformation are then calculated to characterize the toughening effect. Though the main ingredients of transformation toughening have been successfully captured by these theoretical models, there are still many debates on this topic till now. For example, Chen [17] studied transformation toughening in zirconia ceramics with the shear/dilatation criterion. Rauchs et al. [30] found that the shear-dilatant criterion developed from multi-axial compression tests [15,17,29] does not remain valid for tensile stress states. Besides that, the shear-dilatant criterion [15,17,29], as well as the other theoretical models [12,13,14,28], predicts that the shape of the transformation zone is equidimensional. However, it was observed in some tetragonal zirconia polycrystal (TZP) samples or zirconia composites that the autocatalytic transformation occurred and led to formation of a long finger-like transformation zone [4,5,6,7], which cannot be predicted by any macroscopic transformation criteria. In order to properly characterize the geometric features of the transformation zone around the crack tip as well as its toughening effect on crack propagation, the stress-induced phase transformation around the crack tip should be properly characterized [23,30], which is only possible by resorting to microscopic models.

The phase field (PF) method is a mesoscale numerical approach which has been proved to be effective in tracking the microstructural evolution process in materials [31]. The PF method was widely applied to study solid state phase transformations [32], grain growth [33], dislocation [34,35,36] and crack propagation [37,38,39,40], etc. In the past decades, several PF models have been developed to characterize the phase patterns in the process of stress-induced martensitic phase transformation [41,42,43]. In particular, Mamivand et al. [44,45,46] developed a PF model to characterize the phase transformation process in tetragonal zirconia, which was later extended to study the temperature effect and grain size effect on the mechanical responses of TZPs under tension and compression [47]. Sun et al. [48,49] developed PF models to study the thermomechanical responses of shape memory alloys (SMAs).

On the other hand, the PF method for fracture based on the smeared crack model has been proven to be a promising numerical approach to model crack propagation with complex patterns [37,38,39,40,50,51,52,53,54,55]. In the framework of PF model, the coupling of multi-physical processes can be naturally considered. To name a few, Abdollahi and Arias [52,53] studied crack propagation in ferroelectric materials with consideration of ferroelectric domain switching. Schmitt et al. [54] developed a combined PF model to investigate phase transformation and damage in iron. In our previous work [39], a coupled PF model was developed to study the fracture behavior of single crystalline tetragonal zirconia [39], which was later extended to investigate intergranular microcracking in the hydrothermal degradation process [40]. Very recently, Moshkelgosha and Mamivand [56] studied crack growth and deflection in single crystal tetragonal zirconia with PF method. In this paper, the coupled PF model presented in our previous work [39,40] is amended by using the single well potential PF model of fracture, which is more consistent with Griffith theory for brittle fracture. The improvements of the PF model proposed in our previous study are also included in the modified coupled PF model. After that, the stress-induced phase transformation process around a static crack tip is characterized with PF simulations. The finite size and shape of the transformation around the crack tip are firstly predicted on the mesoscale. The influences of the external load and ambient temperature on the size and shape of the transformation zone are discussed. The influence of phase transformation on the stress distribution near the crack tip is also examined. After that, crack propagation in TZP is studied with the new coupled PF model. The toughening effect of phase transformation, the crack propagation, and deflection/branching as well as intergranular microcrack nucleation are systematically investigated. It is shown that the toughness of the grain boundary (GB) as well as the crystal orientation has significant influences on the topology of crack propagation. In the meanwhile, the magnitude of the external load may also influence the crack propagation path.

The paper is organized as follows: in Section 2, the coupled PF model is introduced. In Section 3, the effects of the applied load, the ambient temperature on the transformation patterns around a static crack are discussed. Then, crack propagation behavior and toughening effect in TZP are studied. The effect of the fracture toughness of the GB, the crystal orientation, and the magnitude of the external load on the topology of crack propagation are studied. Conclusions are drawn in Section 4.

## 2. The Coupled PF Model for Crack Propagation in TZP

In our previous work [47], some amendments have been made on the PF model proposed by Mamivand et al. [44,45,46] to properly characterize the temperature dependence and grain size effect of the t–m transformation. The PF model presented in [47] is combined with the PF model for fracture in this study to study the fracture behavior of TZP. The single well potential PF model for fracture is adopted in this study to better characterize the crack nucleation behavior.

In the PF model, the non-conserved order parameters $\eta_{p}\left(r,t\right)$ stand for the content of the *p*th monoclinic variant and ϕ(r,t) represents the crack phase, where r and *t* are the position vector and time, respectively. The value of the order parameters varies from 0 to 1. For the crack phase, ϕ=0 stands for the fully damaged phase and ϕ=1 stands for the intact phase.

The evolution process of the phase-field variables and the damage parameter can be tracked by solving the phenomenological time-dependent Ginzburg–Landau (TDGL) kinetic equations [57]:

(1) $$\frac{\partial \eta_{p}(r,t)}{\partial t}=-L\frac{\delta F_{tot}}{\delta \eta_{p}(r,t)}$$

(2) $$\frac{\partial \phi (r,t)}{\partial t}=-M\frac{\delta F_{tot}}{\delta \phi (r,t)}$$

where *L* and *M* are the kinetic coefficients of the phase transformation and the mobility parameter of crack propagation, respectively. *F* is the total free energy of the system and can be written as follows [39,40,47]:

(3) $$F_{tot}=F_{ch}+F_{gr}+F_{loc}+F_{el}+F_{gb}$$

where

(4) $$F_{ch}=\int_{V}\left[f(\eta_{1},\eta_{2},\ldots ,\eta_{n})+f_{\mathrm{int}}(\eta_{1},\eta_{2},\ldots ,\eta_{n})\right]dV$$

is the chemical energy for phase transformation, which is composed of the bulk chemical free energy and the interfacial energy. The modified bulk chemical energy is expressed as follows [47]:

(5) $$\begin{array}{l} f(\eta_{1},\eta_{2},\ldots ,\eta_{n})=A(\eta_{1}{}^{2}+\eta_{2}{}^{2}+\cdots +\eta_{n}{}^{2})+(4\Delta G-2A)\\ \times (\eta_{1}{}^{3}+\eta_{2}{}^{3}+\cdots +\eta_{n}{}^{3})+(A-3\Delta G){\left(\eta_{1}{}^{2}+\eta_{2}{}^{2}+\cdots +\eta_{n}{}^{2}\right)}^{2} \end{array}$$

where ΔG is the temperature-dependent chemical driving force [44]. The polynomial satisfies the following conditions [47]:

(6) $$\left\{ \begin{array}{l} f^{\prime }\left(0\right)=f^{\prime }\left(1\right)=0\\ f\left(1\right)-f\left(0\right)=\Delta G\\ f^{\prime \prime }\left(0\right)=2A>0\\ f^{\prime \prime }\left(1\right)=2A-12\Delta G>0 \end{array}\right.$$

The energy barrier for phase transformation can be written as follows:

(7) $$G_{barrier}=\frac{A^{3}\left(A-4\Delta G\right)}{16{\left(A-3\Delta G\right)}^{3}}$$

It is noted that $G_{barrier}$ is positively correlated to A. In our previous work, it is suggested that A takes the following form [47]:

(8) $$A=\left\{ \begin{array}{ll} \frac{C}{1+\frac{k_{1}}{T_{0}}\left(T_{0}-T\right)} & \mathrm{when}\;\left(T\leq T_{0}\right)\\ C\left[1+\frac{k_{2}}{T_{0}}\left(T-T_{0}\right)\right] & \mathrm{when}\;\left(T>T_{0}\right) \end{array}\right.$$

where C is a material constant characterizing the energy barrier at the equilibrium temperature and where $T_{0}$, $k_{1}$, and $k_{2}$ are dimensionless parameters.

The interfacial energy between different phases can be written as follows [44]:

(9) $$f_{\mathrm{int}}(\eta_{1},\eta_{2},\ldots ,\eta_{n})=\frac{1}{2}\sum_{p=1}^{n}\beta_{ij}\left(p\right)\nabla_{i}\eta_{p}\nabla_{j}\eta_{p}$$

where $\beta_{ij}(p)$ is the gradient energy coefficient tensor. Levitas and Javanbakht [58,59], and Javanbakht and Barati [60] suggest that the interfacial energy can be written as follows:

(10) $$K_{n}=\lambda +\frac{2}{n}\mu$$

where the parameter *b* (0≤b≤1) is introduced to adjust the ratio of the interfacial energy at the M–M interface to that at the A–M interface. Referring to Equations (5) and (10), we obtain the following:

(11) $$\begin{array}{l} F_{ch}=\int_{V}A(\eta_{1}{}^{2}+\eta_{2}{}^{2}+\cdots +\eta_{n}{}^{2})+(4\Delta G-2A)\\ \times (\eta_{1}{}^{3}+\eta_{2}{}^{3}+\cdots +\eta_{n}{}^{3})+(A-3\Delta G){\left(\eta_{1}{}^{2}+\eta_{2}{}^{2}+\cdots +\eta_{n}{}^{2}\right)}^{2}\\ +\frac{\beta }{2}\left(\sum_{p=1}^{n}{\left(\nabla \eta_{p}\right)}^{2}+b\sum_{i=1}^{n}\sum_{j=1,i\neq j}^{n}\nabla \eta_{i}\cdot \nabla \eta_{j}\right)dV \end{array}$$

$F_{gr}$ and $F_{loc}$ are the gradient energy and the local free energy of the crack phase, respectively. Referring to Kuhn and Müller [50], we have the following:

(12) $$F_{gr}=G_{c}\int_{V}\kappa {\left|\nabla \phi \right|}^{2}dV$$

(13) $$F_{loc}=G_{c}\int_{V}\frac{{\left(1-\phi \right)}^{2}}{4\kappa }dV$$

where $G_{c}$ is the fracture energy and κ controls the width of the transition area between the intact phase and the broken phase.

The elastic strain energy of the material can be written as follows:

(14) $$F_{el}=\frac{1}{2}\int_{V}C_{ijkl}\left(\eta_{p},\phi \right)\varepsilon_{ij}^{el}\varepsilon_{ij}^{el}dV$$

where $C_{ijkl}$ is the fourth-order elastic tensor; $\varepsilon_{ij}^{el}$ is the elastic strain which is the difference between the total strain $\varepsilon_{ij}^{tot}$ and the stress free strain $\varepsilon_{ij}^{0}$ [42,44]:

(15) $$\varepsilon_{ij}^{el}=\varepsilon_{ij}^{tot}-\varepsilon_{ij}^{0}=\frac{1}{2}\left(u_{i,j}+u_{j,i}\right)-\sum_{p}\varepsilon_{ij}^{00}\left(p\right)\eta_{p}^{2}$$

In Equation (15), $\varepsilon_{ij}^{00}$ is the transformation strain. In the PF model, the elastic tensor is a smooth function of the phase variables $\eta_{p}$ [44] and the crack phase ϕ [37]:

(16) $$C\left(\eta_{p},\phi \right)=h\left(\phi \right)C\left(\eta \right)$$

where h(ϕ) is the interpolation function. In this study, we assume h(ϕ) takes the following form:

(17) $$h\left(\phi \right)=\delta +\left(1-\delta \right)\left(-2\phi^{3}+3\phi^{2}\right)$$

where δ is a small value which can retain a low residual stiffness in the fully damaged phase to avoid numerical issues. C(η) is the elastic tensor which is related to the phase variables by the following [44]:

(18) $$C\left(\eta \right)=P(\sum_{p=1}^{n}\eta_{p})C^{M}+((1-P(\sum_{p=1}^{n}\eta_{p}))C^{T}$$

where the superscripts *M* and *T* represent the monoclinic and tetragonal phases correspondingly. The interpolation function P(η) describes the transition of the modulus between the tetragonal and monoclinic phases and takes the following form [46]:

(19) P(η)=η

To simulate the t–m transformation in polycrystals, the local tensorial quantities in different grains are transferred to the global coordinate. The global transformation strain $\varepsilon_{ij}^{G00}$ and elastic tensor $C_{ijkl}^{G}$ can be written as follows [61]:

(20) $$\varepsilon_{ij}^{G00}=R_{ik}R_{jl}\varepsilon_{kl}^{00}$$

(21) $$C_{ijkl}^{G}=R_{im}R_{jn}R_{kr}R_{ls}C_{mnrs}$$

where $R_{ij}$ is the rotation tensor [61].

In this paper, the material properties are assumed to be isotropic for simplification, and the isotropic elastic energy is written as follows:

(22) $$F_{el}=\int_{V}h\left(\phi \right)\left[\frac{\lambda }{2}{\left(\varepsilon_{ii}^{el}\right)}^{2}+\mu \varepsilon_{ij}^{el}\varepsilon_{ij}^{el}\right]dV$$

where $\lambda =\frac{2\mu \nu }{1-2\nu }$ and $\mu =\frac{E}{2\left(1+\nu \right)}$ are Lamé constants. To avoid fracture under the compression state, the elastic energy is split into two parts [62]:

(23) $$F_{el}=\int_{V}\frac{K_{n}}{2}{\left(\varepsilon_{ii}^{el}\right)}_{-}^{2}+h\left(\phi \right)\left[\frac{K_{n}}{2}{\left(\varepsilon_{ii}^{el}\right)}_{+}^{2}+\mu \left(\varepsilon_{ij}^{el}-\frac{1}{n}\varepsilon_{kk}^{el}\delta_{ij}\right)\left(\varepsilon_{ij}^{el}-\frac{1}{n}\varepsilon_{ll}^{el}\delta_{ij}\right)\right]dV$$

where

(24) $${\left(\varepsilon_{ii}\right)}_{-}={\langle tr\left(\varepsilon \right)\rangle }_{-}=\left\{ \begin{array}{ll} tr\left(\varepsilon \right) & \mathrm{if}\;tr\left(\varepsilon \right)<0\\ 0 & \mathrm{else} \end{array}\right.$$

and

(25) $${\left(\varepsilon_{ii}\right)}_{+}={\langle tr\left(\varepsilon \right)\rangle }_{+}=\left\{ \begin{array}{ll} tr\left(\varepsilon \right) & \mathrm{if}\;tr\left(\varepsilon \right)\geq 0\\ 0 & \mathrm{else} \end{array}\right.$$

Herein,

(26) $$K_{n}=\lambda +\frac{2}{n}\mu$$

denotes the n-dimensional bulk modulus. With such a treatment, only the tensile and deviatoric parts of the elastic energy contribute to crack nucleation and propagation.

The GBs are assumed to possess finite width [63,64]. An extra GB energy term is introduced to describe the constraint effect of GBs on the phase transformation [47,48,65], which takes the following form [47]:

(27) $$F_{gb}=\int_{V}\alpha_{0}\sum_{p=1}^{n}\eta_{p}^{2}dV$$

where $\alpha_{0}$ is positive and only nonzero in the GB zone and $F_{gb}$ stands for the excess energy required for the phase transformation to traverse the GB.

By substituting Equations (12)–(14), (23), and (27) into Equations (1) and (2), the TDGL equations become the following:

(28) $$\begin{array}{ll} \frac{\partial \eta_{p}(r,t)}{\partial t}= & L\{\beta \left(\nabla^{2}\eta_{p}(r,t)+b\sum_{q=1,q\neq p}^{n}\nabla^{2}\eta_{q}(r,t)\right)\\ & -\left(2\left(A+\alpha_{0}\right)\eta_{p}+3\left(4\Delta G-2A\right)\eta_{p}^{2}+4\left(A-3\Delta G\right)\eta_{p}\left(\sum_{p=1}^{n}\eta_{p}^{2}\right)\right)+\frac{\delta F_{el}}{\delta \eta_{p}\left(r,t\right)}\} \end{array}$$

(29) $$\begin{array}{ll} \frac{\partial \phi (r,t)}{\partial t}= & M\{2G_{c}\kappa \nabla^{2}\phi -\frac{G_{c}}{2\kappa }(\phi -1)\\ & +h^{\prime }\left(\phi \right)\left[\frac{K_{n}}{2}{\left(\varepsilon_{ii}^{el}\right)}_{+}^{2}+\mu \left(\varepsilon_{ij}^{el}-\frac{1}{n}\varepsilon_{kk}^{el}\delta_{ij}\right)\left(\varepsilon_{ij}^{el}-\frac{1}{n}\varepsilon_{ll}^{el}\delta_{ij}\right)\right]\} \end{array}$$

where

(30) $$\begin{array}{ll} \frac{\delta F_{el}}{\delta \eta_{p}\left(r,t\right)}= & -2K_{n}{\left(\varepsilon_{ii}^{el}\right)}_{-}\cdot \varepsilon_{jj}^{00}\left(p\right)\eta_{p}+h\left(\phi \right)[-2K_{n}{\left(\varepsilon_{ii}^{el}\right)}_{+}\cdot \varepsilon_{jj}^{00}\left(p\right)\eta_{p}\\ & +4\mu \left(\varepsilon_{ij}^{el}-\frac{1}{n}\delta_{ij}\varepsilon_{kk}^{el}\right)\left(-\varepsilon_{ij}^{00}\left(p\right)\eta_{p}+\frac{1}{n}\delta_{ij}\varepsilon_{ll}^{00}\left(p\right)\eta_{p}\right)] \end{array}$$

The TDGL equations are coupled with the mechanical equilibrium equations to solve the displacements of the system:

(31) $$\begin{array}{l} \sigma_{ij,j}=0\Rightarrow \\ \lambda \left(u_{kk,j}-\sum_{p}\varepsilon_{kk}^{00}\left(p\right)\frac{\partial }{\partial r_{j}}\left(\eta_{p}^{2}\left(r\right)\right)\right)\delta_{ij}+2\mu \left(\frac{1}{2}\left(u_{k,l}+u_{l,k}\right)-\sum_{p}\varepsilon_{kl}^{00}\left(p\right)\frac{\partial }{\partial r_{j}}\left(\eta_{p}^{2}\left(r\right)\right)\right)=0 \end{array}$$

Equations (28), (29), and (31) are the governing equations of the coupled PF model. In the solution process, the external load is considered as the boundary conditions of the computational domain.

## 3. Results and Discussion

**Figure 1:**
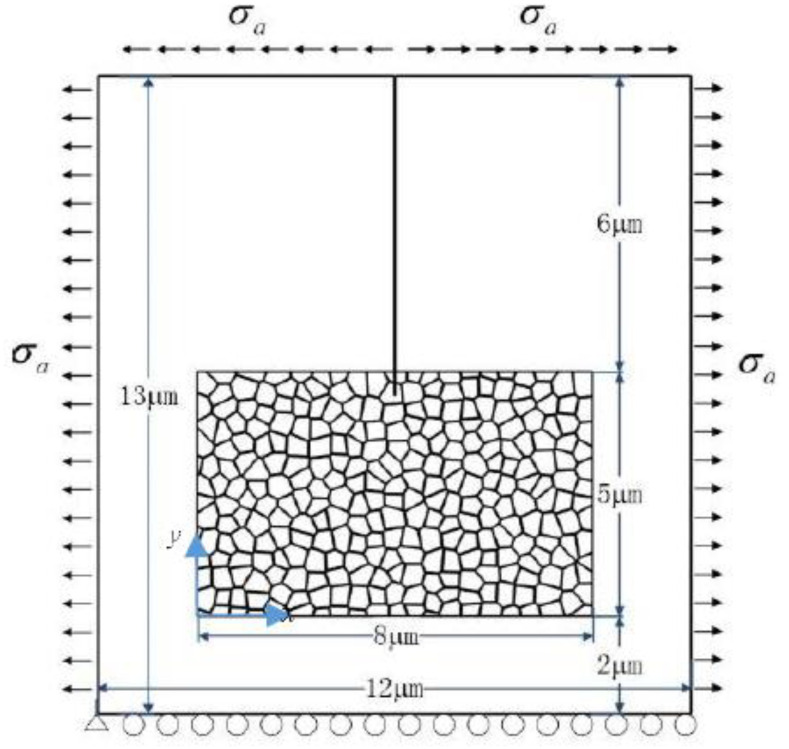
The polycrystal model and the boundary conditions.

A 2D polycrystal model with an edge crack is built up to investigate the fracture behavior of TZP, as shown in Figure 1. The geometry of the polycrystal is firstly generated with the voronoi method. After that, some modification has been made manually so that the GBs possess finite width. The polycrystal model occupies a rectangle domain (8 μm×5 μm) and is embedded in an untransformable matrix (12 μm×13 μm). The initial crack length is 6.5 μm. The average grain size of the polycrystal model is 350 nm. The crystal orientations of the grains are randomly selected and fall in the range: $0^{{}^{\circ}}–180^{{}^{\circ}}$. The GBs possess a finite width of 5 nm. This value is selected to balance the physical reality and the computation cost. The loading and displacement boundary conditions are illustrated in Figure 1. In the numerical simulation, the zero flux conditions are enforced on the boundaries:

(32) $$n\cdot \nabla \eta_{p}=0,\;p=1,2$$

(33) n⋅∇ϕ=0

where n is the outward normal of the boundary. A minor disturbance ($1\times 10^{-6}$) for $\eta_{1}$ and $\eta_{2}$ is assigned in the grains so that the transformation can be initiated under external loading.

In this study, the material properties are assumed to be homogeneous and isotropic to simplify the analysis. The material properties and the values of the other parameters in the PF model are listed in Table 1 and Table 2, respectively. The PF model is implemented with COMSOL Multiphysics. The FE mesh is carefully refined so that there are 3–4 layers of elements across the GBs. The convergence requirement of the mesh size is checked in the numerical studies.

### 3.1. Transformation Zone around a Static Crack

Under external loading, phase transformation is initiated around the crack tip. In this section, the evolution of the phase patterns is directly characterized with PF simulations. The geometric features of the transformation zone around a static crack is examined. The effects of the external loading and ambient temperature on the size and shape of the phase transformation zone are discussed. The stress distribution near the crack tip before and after phase transformation is exhibited to show the toughening effect.

**Figure 2:**
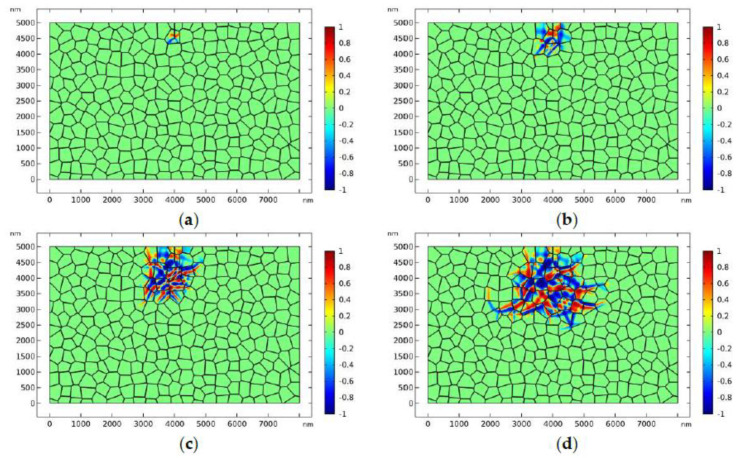
Stress-induced phase transformation around the static crack tip, where $\sigma_{a}=90\;\mathrm{MPa}$ and *T* = 1360 K: the transformation patterns are exhibited by plotting $\eta_{1}-\eta_{2}$. Figure 2(**a**–**d**) correspond to times: 0.01, 0.05, 0.2, and 0.4 μs, respectively.

Figure 2 shows the evolution of the phase transformation around the edge crack where the applied load is $\sigma_{a}=90\;\mathrm{MPa}$ and the simulation temperature is *T* = 1360 K. Figure 2 shows that the phase transformation initiates from the tip of the crack, which is a distinct manifestation of stress-induced phase transformation in the high-stress concentration area. The phase transformation propagates into the adjacent grains and reaches the equilibrium state (Figure 2d). It can be found that there exists a phase transformation zone around the crack tip. At the fringe of the transformation zone, monoclinic variants are present, which is the evidence of autocatalytic transformation in TZP. Overall, the transformation zone is equidimensional with some elongated finger-like monoclinic phases at the fringe of the phase transformation zone. It should be mentioned that this is the first mesoscale model that can successfully predict the shape and size of the transformation zone around the crack tip.

**Figure 3:**
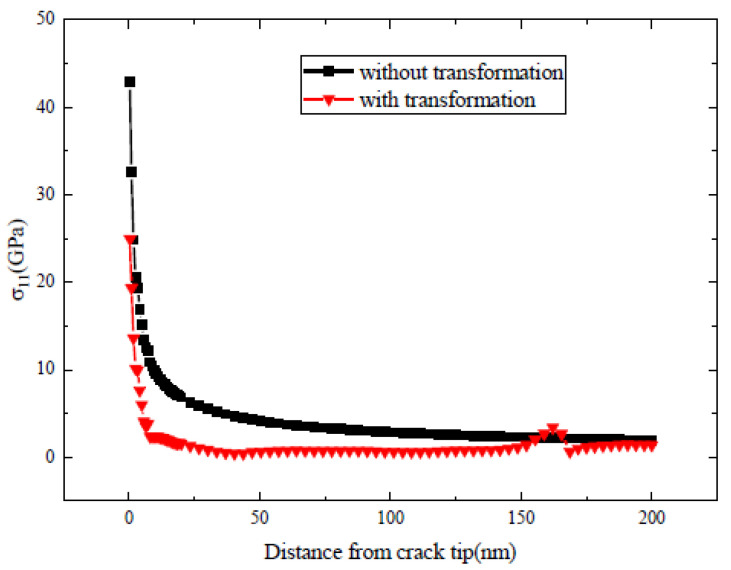
Distribution of the stress component $\sigma_{11}$ in front of the crack tip for the cases where the phase transformation is considered or ignored.

As the load is axisymmetric with respect to the crack plane, the crack tends to propagate in mode I if the phase transformation is not considered. To show the effect of phase transformation on crack propagation, the distribution of the horizontal normal stress $\sigma_{11}$ in front of the crack tip is plotted in Figure 3. In Figure 3, the distribution of $\sigma_{11}$ in front of the crack tip is compared for the cases where the phase transformation is considered or ignored (linearly elastic case). Figure 3 shows that, overall, the phase transformation has the tendency to decrease the stress near the crack tip. As a result, crack propagation will be retarded due to the existence of the phase transformation zone. That is to say, the phase transformation can produce toughening effect on the initial static crack. It is noted that the toughening effect of phase transformation on the initial crack has been also predicted by some theoretical models [12,13,39]. In Figure 3, it is noted that, occasionally, the horizontal normal stress with consideration of phase transformation may exceed that for the linearly elastic case. At that specific location, the monoclinic variants intersect with the grain boundary and cause stress concentration.

**Figure 4:**
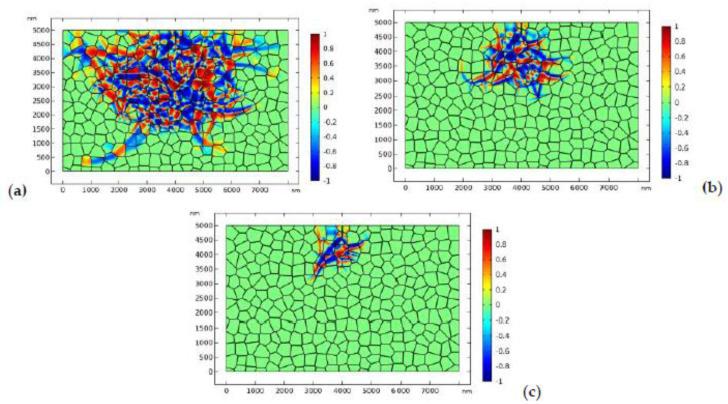
The equilibrium phase patterns around the crack tip at different ambient temperatures: (**a**) T=1340 K; (**b**) T=1360 K; and (**c**) T=1380 K. The applied load is $\sigma_{a}=90\;\mathrm{MPa}$.

**Figure 5:**
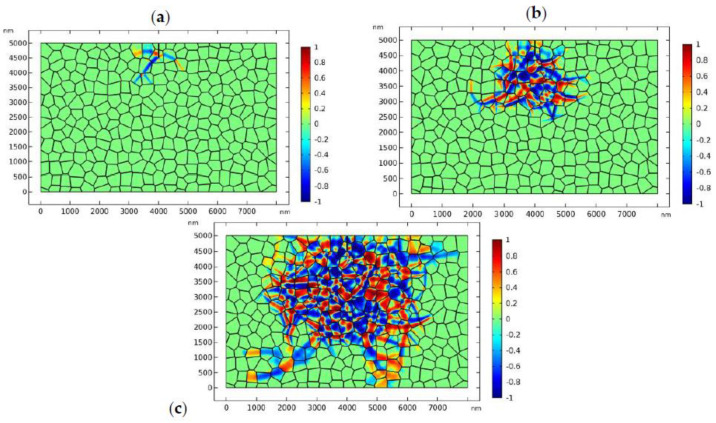
The equilibrium phase patterns around the crack tip under different loads: (**a**) $\sigma_{a}=60\;\mathrm{MPa}$; (**b**) $\sigma_{a}=90\;\mathrm{MPa}$; and (**c**) $\sigma_{a}=120\;\mathrm{MPa}$. The ambient temperature is T=1360 K.

In Figure 4 and Figure 5, the geometry features of the transformation zone under different loads and ambient temperatures are presented. In Figure 4, the load keeps constant, i.e., $\sigma_{a}=90\;\mathrm{MPa}$, while the temperature changes from 1340 K to 1380 K. The equilibrium patterns of the phase transformation around the crack tip are plotted in Figure 4a–c. It can be found that the size of the transformation zone decreases as the temperature increases. Referring to Equation (8), the energy barrier generally increases with temperature. Thus, the critical stress for phase transformation becomes higher as temperature increases. As a result, the size of the transformation zone tends to decrease with the ambient temperature. This phenomenon is consistent with previous studies. For example, Becher and Swain [9], and Rose and Swain [11] have confirmed by experiments that the size of the transformation zone is negatively correlated with temperature.

The equilibrium phase patterns around the crack tip under different loads are shown in Figure 5. In Figure 5a–c, the applied loads are 60 MPa, 90 MPa, and 120 MPa, respectively. The simulation temperature is 1360 K. As shown in Figure 5, it can be easily found that the size of the transformation zone is positively correlated to the applied load. Chen and Reyes Morel [15] proposed a formula based on the shear/dilatation criterion to predict the transformation zone. Yu and Shetty [4] provided a simplified formula to predict the characteristic length of the transformation zone. Both models suggest that the size of the transformation zone is proportional to the square of the applied stress intensity factor (SIF), i.e., $r\propto K_{Ia}^{2}$, where $K_{Ia}$ is the applied SIF. Since $K_{Ia}\propto \sigma_{a}$, it can be deduced that $r\propto \sigma_{a}^{2}$. At the mesoscale, this relation may be not accurate to describe the relation between the size of the transformation zone and the external load. However, it still shows that the transformation size may increase dramatically with the external load. Compared to the size of the transformation zone shown in Figure 5, it can be found that the simulation results are in accordance with this conclusion.

After adjusting the crystal orientations of the polycrystal, the conclusions drawn above are still valid. The detailed numerical results are omitted here. This shows that the PF model developed in this study can be effective numerical tool to characterize the size and shape of the phase transformation zone around the crack tip, which is a prerequisite to further studying the toughening effect of phase transformation on growing cracks.

### 3.2. Crack Propagation in TZP

**Figure 6:**
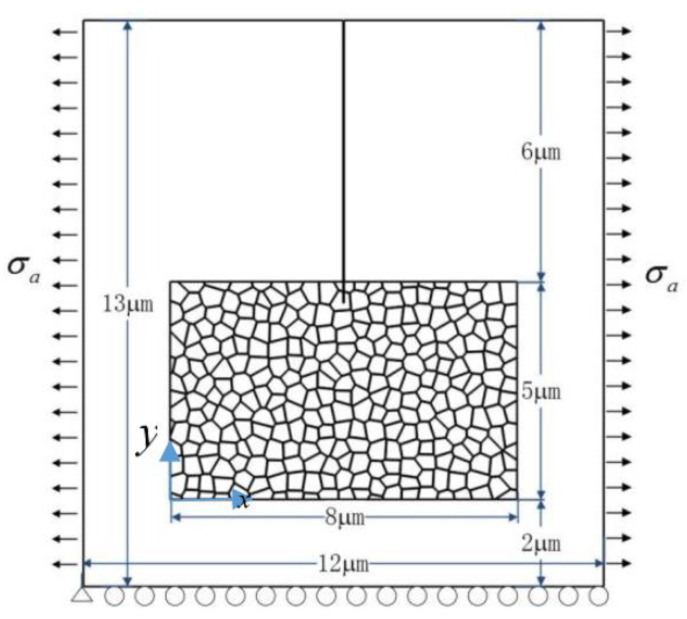
The polycrystal model under tensile load.

In TZP, crack propagation is accompanied with the tetragonal to monoclinic phase transformation. The phase transformation will produce a toughening effect on crack propagation. On the other hand, as the stress field is perturbed by the complex phase patterns, the crack may experience deflection [9,67] or branching [68,69,70] in the stress driven propagation process. Crack deflection and branching may also contribute to the toughening effect. In this section, crack propagation in TZP is studied with PF simulations. We assume that the intrinsic fracture toughness of TZP is uniform in all the grains and is assumed to be $1.2\;\mathrm{MPa}\sqrt{m}$. Usually, the GB zone possesses lower fracture toughness. In the numerical studies, the fracture toughness of the GB zone is tuned to study its effect on crack propagation. A tensile load is applied at the side boundaries, as shown in Figure 6. The dimension of the numerical model and the crack length are illustrated in Figure 6. In the numerical studies, two different groups of crystal orientations are considered, which are denoted crystal orientation I and II in the following discussions, respectively.

**Figure 7:**
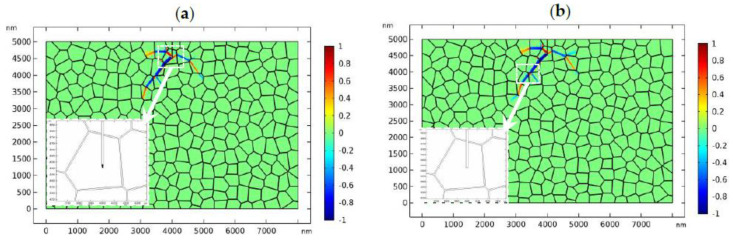
The evolution of the phase transformation and crack propagation in tetragonal zirconia polycrystal (TZP) (crystal orientation I) where the applied load is 110 MPa (**a**,**b**) at times 0.4 μs and 2 μs, respectively.

The critical load for crack propagation in linearly elastic material is determined by numerical simulations, which is around 98 MPa. When phase transformation is present, 98 MPa is insufficient to initiate the crack propagation due to the toughening effect of phase transformation (Figure 3). In Figure 7, the applied load increases to 110 MPa. The fracture toughness of the GB zone is assumed to be the same as that in the bulk material, i.e., $1.2\;\mathrm{MPa}\sqrt{m}$. Figure 7 shows the evolution of the phase pattern and the crack phase. In Figure 7a, an initial perturbation of the crack phase is specified at the crack tip. The evolution of the crack phase is terminated for 0.4 μs until the phase evolution around the crack tip has reached the equilibrium state (Figure 7a). After that, the crack phase and the phase transformation are allowed to evolve simultaneously. The simulation results are shown in Figure 7b. It should be noted that the PF model for fracture adopted in this paper allows crack healing. In Figure 7b, the phase pattern doesn’t change much compared with Figure 7a. However, the crack phase experiences degradation, which shows the toughening effect of the phase transformation.

**Figure 8:**
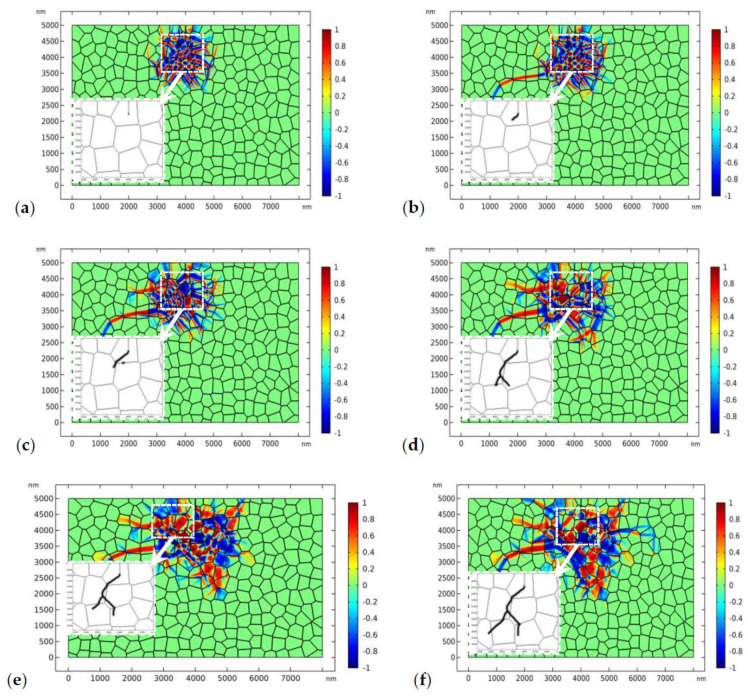
The evolution of the phase transformation and crack propagation in TZP (crystal orientation I) with “strong” grain boundaries (GBs) (plots represent $\eta_{1}-\eta_{2}$, the crack is displayed in the amplified window separately), where the applied load is 180 MPa: (**a**,**b**) at times 0.4 and 6 μs, respectively, where the applied load is 170 MPa, and (**c**–**f**) at times 7.0, 7.5, 8.0, and 10.0 μs, respectively.

The results shown in Figure 7 implies that a higher load is needed to drive the crack to propagate forward. In Figure 8, the applied load increases to 170 MPa (Figure 8a,b) and 180 MPa (Figure 8c–f) respectively. We assume that the fracture toughness of the GB zone is the same as that in the bulk material, i.e.,$1.2\;\mathrm{MPa}\sqrt{m}$, which is referred to as “strong GB” in the following discussions. Figure 8a,b shows the crack propagation and phase evolution when the tensile load is 170 MPa. The crack phase is exhibited by deleting the elements with ϕ<0.5. To show the crack propagation path more clearly, the crack phase is shown separately in the amplified window. The evolution of the crack phase is terminated for 0.4 μs until the phase evolution around the initial crack tip has reached the equilibrium state (Figure 8a). After that, the crack phase and the phase transformation patterns are allowed to evolve simultaneously. As shown in Figure 8b, the crack deflected from its initial path due to the perturbation of the stress field by the monoclinic variants. A similar phenomenon has been observed in previous experimental works [9,67]. After a short period of propagation, the crack growth has been totally shielded by the phase transformation. It is noted that, in linearly elastic materials, the crack will propagate in mode I with accelerating speed. The termination of crack propagation in Figure 8b is clearly due to the toughening effect of phase transformation.

In order to drive the crack to propagate forward, the load increases to 180 MPa in Figure 8c–f. Figure 8c shows the nucleation of a microcrack in the GB zone where the monoclinic variant intersects with the GB. This transformation-induced intergranular microcracking has been systematically discussed in our previous study [40]. As crack healing is allowed in the current PF model, the microcrack is healed after a short period of time as the main crack propagates forward. In Figure 8d–f, the crack branches into two parts. It should be mentioned that crack branching has been observed in some experimental works [68,69,70]. It can be observed that the crack propagates transgranularly. In the meanwhile, the size of the transformed zone has expanded a lot as the crack propagates forward.

**Figure 9:**
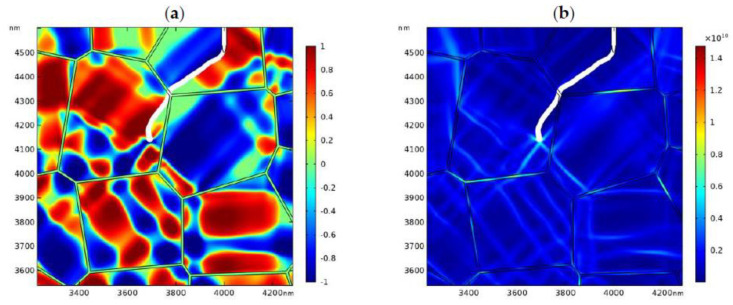
The local phase pattern (**a**) and the local von Mises stress field (**b**) just before crack branching (Figure 8d).

The distribution of the phase patterns and the von Mises stress just before crack branching (Figure 8d) are shown in Figure 9. It can be found that there exist phase boundaries in front of the crack tip (Figure 9a) which cause an “X”-like stress distribution pattern (Figure 9b). This explains the physical origin of crack branching in Figure 8d. Both the crack deflection and branching are due to the stress perturbation brought about by the complex phase patterns.

**Figure 10:**
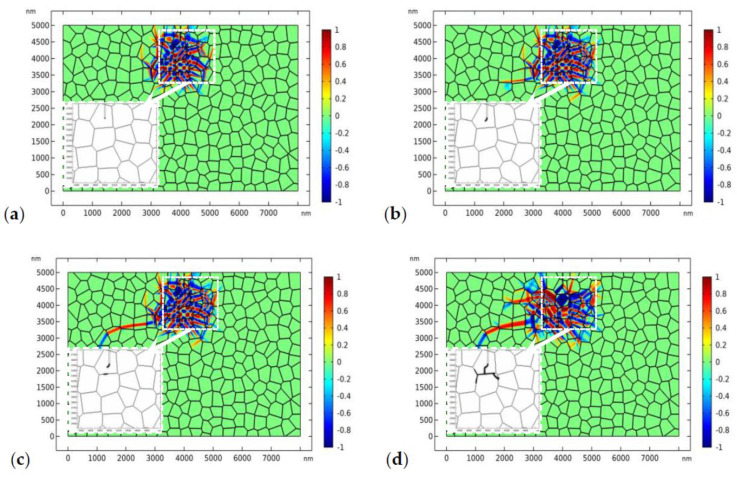

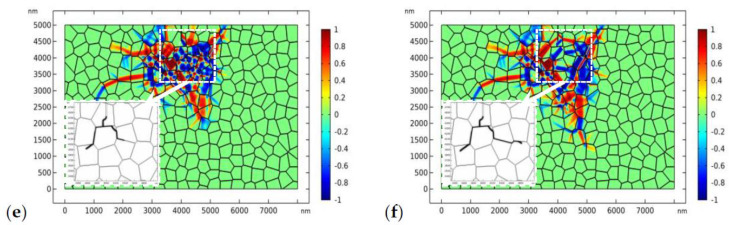
The evolution of the phase transformation and crack propagation in TZP (crystal orientation I) with “weak” GBs (plots represent $\eta_{1}-\eta_{2}$, and the crack is displayed in the amplified window separately), where the applied load is 170 MPa: (**a**–**f**) at times 0.4, 0.8, 1.2, 1.6, 2.0, and 2.8 μs, respectively.

**Figure 11:**
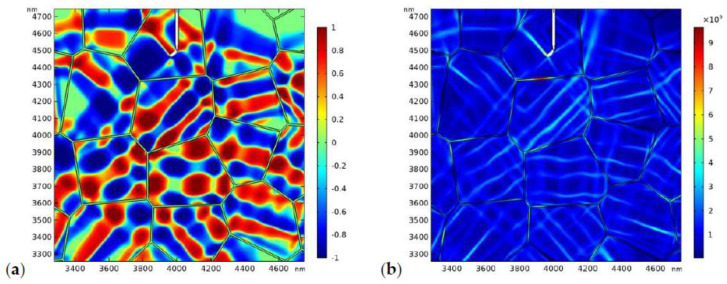
The local phase pattern (**a**) and the local von Mises stress field (**b**) before the microcrack nucleation (corresponding to Figure 10b).

In the second case, we consider more practical situation where the GB zone possesses lower fracture toughness. The fracture toughness of the GB zone is assumed to be $0.5\;\mathrm{MPa}\sqrt{m}$. The applied stress is 170 MPa. The evolution of the phase pattern and the crack propagation are shown in Figure 10. Figure 10a shows the phase pattern around the initial crack (before crack propagation). In Figure 10b, the crack starts to propagate forward. Again, crack deflection can be observed. In Figure 10c, a microcrack starts to nucleate at the GB zone. The local phase pattern and the distribution of von Mises stress before microcrack nucleation are shown in Figure 11. As shown in Figure 11b, high local stress is accumulated at the GB zone in front of the crack tip, which is due to the intersection of the monoclinic variant and the GB. Finally, a microcrack nucleates from the intersection point. As the GB zone possess lower fracture toughness, the nucleated microcrack tends to propagate intergranularly and may penetrate into the bulk grain occasionally (Figure 10d). Finally, the main crack converges with the intergranular microcrack (Figure 10d–f). It is noted that intergranular microcracks around the main crack and crack-bridging have been observed in experimental works [11,69,70,71,72].

The converged crack tends to propagate intergranularly. Crack healing is also found during the evolution of the crack phase. The phase patterns evolve simultaneously along with crack propagation. Some of the monoclinic variants have degraded behind the crack tip due to the variation of the stress state. Comparing Figure 9 and Figure 10, we can find that, for TZP with “strong” GBs, the crack tends to propagates transgranularly while, for TZP with “weak” GBs, the crack is more likely to grow intergranularly. In the latter case, it is easier for the intergranular microcrack to nucleate in the GB zone. Intergranular microcracks have been observed in many experimental works [11,71,72,73]. In addition, for crack propagation in TZP with a “strong” GB, higher external load (180 MPa) is needed while, in TZP with a “weak” GB, 170 MPa is sufficient to drive the intergranular crack propagation. This implies that the toughness of the GB zone may have a close relation with the strength of the material.

**Figure 12:**
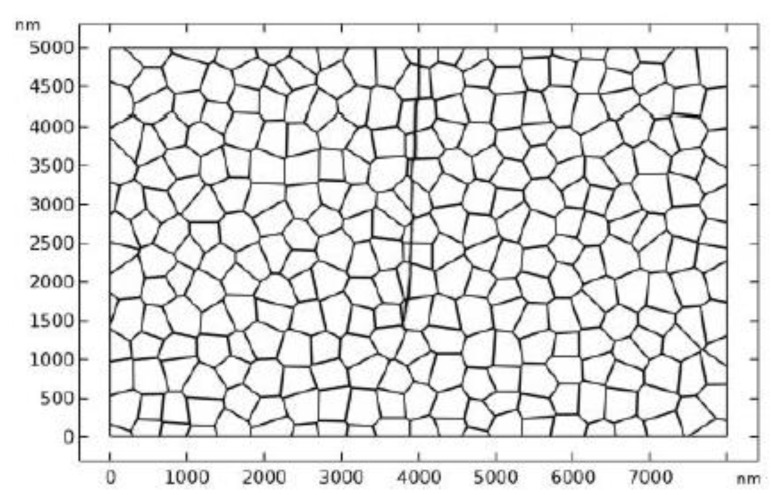
Crack propagation in linearly elastic material with “weak” GBs.

In comparison, crack propagation in linearly elastic material with “weak” GBs is shown in Figure 12. As the fracture toughness of the GB area is lower, the crack phase starts to evolve on the GBs under external loading though the evolution does not lead to a fully damaged crack phase. As the modulus is related with the order parameter of the crack phase (Equation 23), the crack is no longer in mode I due to the loss of material symmetry. The crack propagates transgranularly and experiences deflection each time it passes the GB. A similar crack propagation pattern was observed in a 16Ce-TZP sample where no transformation around the crack was detected with an optical microscope [10]. Comparing Figure 10 and Figure 12, it is obvious that the severe deflection of the crack path and the intergranular fracture are outcomes of the perturbation of the stress field by the monoclinic phase.

**Figure 13:**
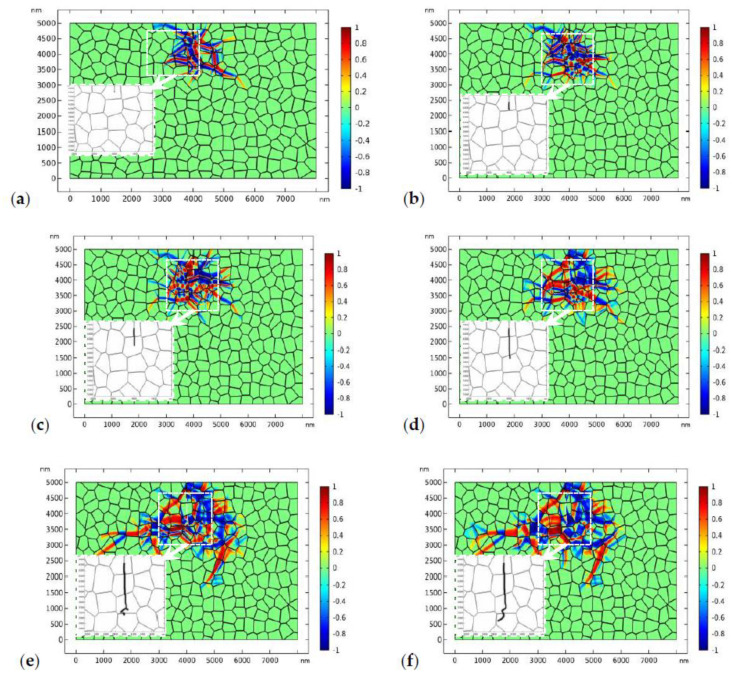
The evolution of the phase transformation and crack propagation in TZP (crystal orientation II) with a “strong” GB (plots represent $\eta_{1}-\eta_{2}$, and the crack phase is displayed in the amplified window separately), where the applied load is 150 MPa: (**a**–**f**) at times 0.4, 3.0, 3.5, 4, 5.0, and 5.5 μs, respectively.

To show the generality of the conclusions drawn above, the crystal orientations of the polycrystal are randomly adjusted, which is referred as crystal orientation II in the following discussions. Again, the effect of the fracture toughness of the GBs on the topology of crack propagation is studied. Firstly, the fracture toughness of the GB zone is assumed to be the same as that in the bulk material, i.e., $1.2\;\mathrm{MPa}\sqrt{m}$. Figure 13 shows the crack propagation and phase evolution where the tensile load is 150 MPa. The evolution of the crack phase is terminated for 0.4 μs until the phase transformation reaches the equilibrium state (Figure 13a). Then, the crack phase and the phase transformation are allowed to evolve simultaneously. The crack propagates with a minor deflection (Figure 13b). In Figure 13b–d, the crack propagates transgranularly and in a nearly straight path. It is noted that the straight propagation path is occasional as it is closely related with the crystal orientations. Crack branching and microcrack nucleation can be observed in Figure 13e. In Figure 13f, one branch of the main crack is healed and the main crack converges with the microcrack which propagates transgranularly into the adjacent grain. As the crack propagates forward, the size of the transformation zone keeps growing. Some of the monoclinic variants have degraded behind the crack tip due to the change of the stress state.

**Figure 14:**
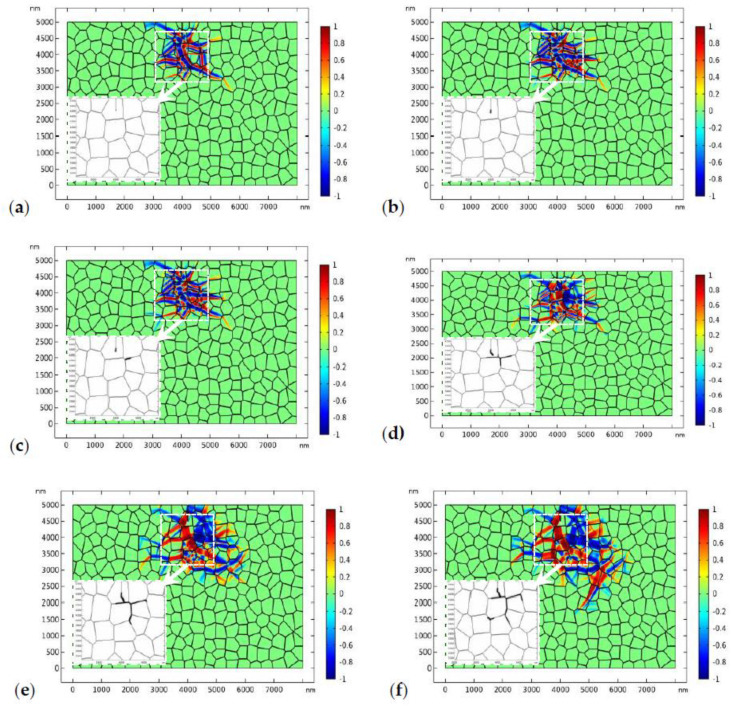
The evolution of the phase transformation and crack propagation in TZP (crystal orientation II) with a “weak” GB (plots represent $\eta_{1}-\eta_{2}$, and the crack is displayed in the amplified window separately), where the applied load is 150 MPa: (**a**–**f**) at times 0.4, 1.0, 1.3, 1.7, 2.0, and 2.5 μs, respectively.

Figure 14 shows the evolution of the phase pattern and the crack propagation when the GB zone possesses lower fracture toughness, i.e., $0.5\;\mathrm{MPa}\sqrt{m}$. The applied stress is 150 MPa. Figure 14a shows the phase pattern before crack propagation. In Figure 14b,c, an intergranular microcrack starts to nucleate in the GB zone as the main crack propagates forward. The microcrack grows intergranularly and the main crack deflects from its initial path (Figure 14d). Then, the main crack converges with the microcrack (Figure 14e). In the crack propagation process, the phase transformation evolves simultaneously. As shown in Figure 14f, another microcrack starts to nucleate at the triple junction. The results presented in Figure 14 show that, for TZP with “weak” GBs, the main crack is more likely to propagate intergranularly as microcracks are easier to nucleate in the GB zone in such cases.

**Figure 15:**
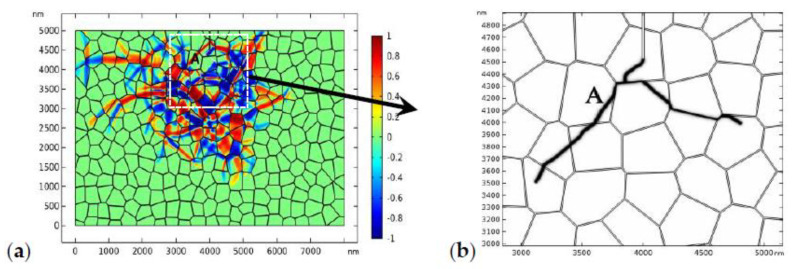
The evolution of the phase transformation (**a**) and crack propagation (**b**) in TZP (crystal orientation I) with a “weak” GB where the fracture toughness of the bulk grains is $1.0\;\mathrm{MPa}\sqrt{m}$ and the fracture toughness of the GB is $0.5\;\mathrm{MPa}\sqrt{m}$. The applied load is 170 MPa.

**Figure 16:**
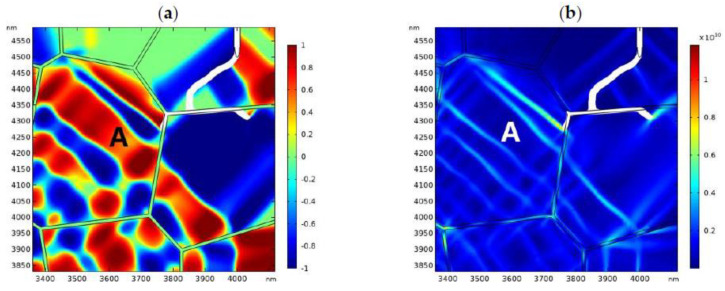
The local transformation pattern (**a**) and the local von Mises stress field (**b**) which correspond to the numerical results presented in Figure 15.

The results presented in Figure 8, Figure 9, Figure 10, Figure 11, Figure 12, Figure 13 and Figure 14 clearly indicate that the crack propagation path is closely related with the relative fracture toughness of the GB with respect to that of the bulk material. To dig it further, in Figure 15, we assume that the bulk grains possesses lower fracture toughness, i.e., $1.0\;\mathrm{MPa}\sqrt{m}$ and the fracture toughness of the GBs is $0.5\;\mathrm{MPa}\sqrt{m}$. The applied load is 170 MPa. The phase transformation patterns and the amplified crack propagation path are shown in Figure 15. In Figure 15, it can be found that the crack still has some tendency to propagate intergranularly but that the ratio of transgranular propagation increases obviously comparing with Figure 10. The local stress distribution in the grain (grain A) where the transgranular crack propagation takes place is shown in Figure 16. As shown in Figure 16, the presence of phase boundaries induces high stress inside grain A, which induced the transgranular fracture.

**Figure 17:**
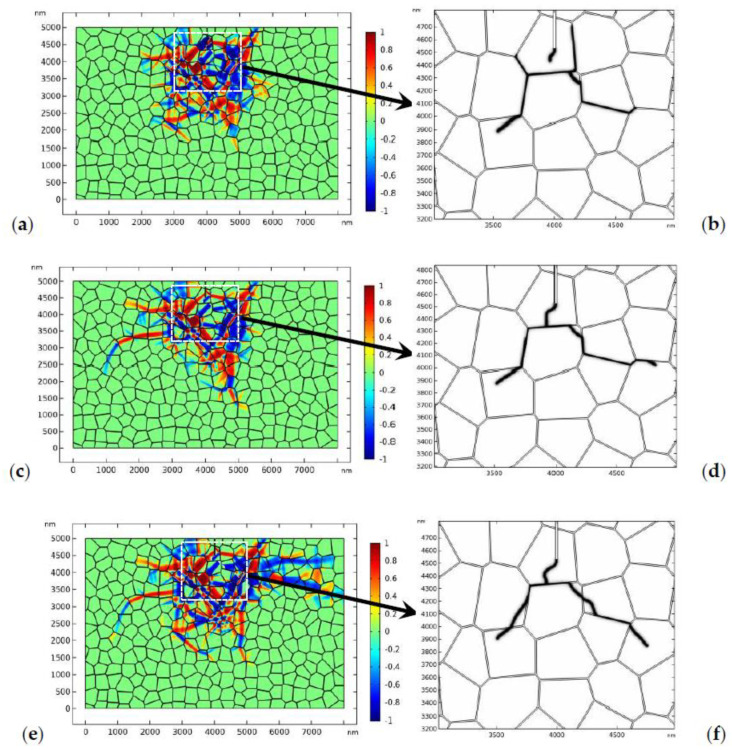
The evolution of the phase transformation and crack propagation in TZP (crystal orientation I) with a “weak” GB where the fracture toughness of the bulk grains is $1.2\;\mathrm{MPa}\sqrt{m}$: The phase pattern and crack propagation path under different applied loads. (**a**,**b**) The load is 160 MPa. The phase patterns and the topology of crack propagation correspond to 3.6 μs. (**c**,**d**) The load is 170 MPa. The phase patterns and the topology of crack propagation correspond to 2.8 μs. (**e**,**f**) The load is 180 MPa. The phase patterns and the topology of crack propagation correspond to 2.2 μs.

In the numerical studies, it is found that, besides the crystal orientation and fracture toughness of the GBs, the magnitude of the external load may also influence the crack propagation path. In Figure 17, the crack propagation paths in TZP with “weak” GBs under different loads, i.e., 160 MPa, 170 MPa, and 180 MPa, are exhibited. As shown in Figure 17, when the applied load is 160 MPa (Figure 17a,b), the crack mainly propagates intergranularly. The main crack experiences deflection. The propagation of the main crack is shielded by the intergranular microcrack before it can converge with the intergranular crack. As the fracture toughness of the GB ($0.5\;\mathrm{MPa}\sqrt{m}$) is much lower than that of the bulk grains, the crack nucleated in the GB zone tends to propagate intergranularly under external load. When the load increases to 170 MPa (Figure 17c,d) or 180 MPa (Figure 17e,f), the main crack can propagate forward and finally converges with the intergranular crack. After convergence, some monoclinic variants behind the crack degraded because of the redistribution of the stress field. Compared to the crack patterns where the load is 160 MPa and 170 MPa, the crack is more likely to propagate transgranularly when the load is 180 MPa. This implies that the external load may also influence the crack growing path. Under higher loads, the crack has the tendency to propagate transgranularly.

## 4. Conclusions

In this paper, the coupled PF model presented in previous work [39,40] is modified to study the fracture behavior of TZP. The modifications include a temperature-dependent free energy functional, the differentiation of the interfacial energy between different phases, an extra energy term to characterize the constraint effect of GBs on phase transformation, and a single well potential PF model for fracture. The modified PF model can directly predict the shape and size of the phase transformation zone around the crack tip where the evolution of the phase patterns is explicitly characterized. The effects of the ambient temperature and the applied load on the transformation zone are discussed. The simulation results are qualitatively consistent with previous experimental observations. After that, crack propagation and transformation toughening effect in TZP are studied with the aid of the coupled PF model. The influences of crystal orientation, the fracture toughness of the GB zone, and the external load on the topology of crack propagation are discussed in detail. The main conclusions are summarized below:

1. The phase evolution process around the crack tip can be directly characterized with the PF model. The shape and size of the phase transformation zone around the crack tip are firstly predicted with the mesoscale PF model.
2. The size of the transformation zone increases dramatically with the external load and decreases with ambient temperature. These results are consistent with the experimental observations [4,15].
3. The phase transformation can decrease the stress around the static crack tip and retards its propagation. The toughening effect of phase transformation on the initial crack has been confirmed by the crack propagation model (Figure 7).
4. For crack propagation in TZP, the phase patterns and the crack evolve simultaneously. The crack may experience deflection or branching due to the perturbation of the stress field by the complex phase patterns.
5. The crystal orientation, the fracture toughness of the GBs, and the external load all have important influences on the topology of crack propagation in TZP. For TZP with strong GBs, the crack tends to propagate transgranularly, while the crack is more likely to propagate intergranularly for TZP with low GB fracture toughness. Under higher loads, the crack has the tendency to propagate transgranularly.

The coupled PF model proposed in this study can act as a robust numerical tool to characterize the fracture behavior and toughening effect of tetragonal zirconia polycrystals.

## Figures and Tables

**Table 1:** Deformation gradient ($F_{ij}$), right stretch tensor ($U_{ij}$), and transformation strain ($\varepsilon_{ij}^{00}$) of the monoclinic variants [66].

Correspondence	$F_{ij}$	$U_{ij}$	$\varepsilon_{ij}^{I00}$	$\varepsilon_{ij}^{\mathrm{II}00}$
ABC	$\left[\begin{array}{ll} 1.0084 & 0.1562\\ 0 & 1.0087 \end{array}\right]$	$\left[\begin{array}{ll} 1.0049 & 0.0761\\ 0.0761 & 1.0180 \end{array}\right]$	$\left[\begin{array}{ll} 0.0049 & 0.0761\\ 0.0761 & 0.0180 \end{array}\right]$	$\left[\begin{array}{ll} 0.0049 & -0.0761\\ -0.0761 & 0.0180 \end{array}\right]$

**Table 2:** Material properties and the other parameters in the phase field (PF) simulation.

$T_{0}$ (K) (equilibrium temperature)	1367
L ($m^{3}/J/s$) (kinetic coefficient)	10
M ($m^{3}/J/s$) (Mobility parameter)	0.1
β (J/m) (gradient energy coefficient)	2.5 × 10^−^
b	0.95
$\alpha_{0}$ (${J/m}^{3}$) (grain boundary energy coefficient)	2 × 10^8^
*C* (${J/m}^{3}$) (energy barrier coefficient)	5.4 × 10^7^
$k_{1}$	13
$k_{2}$	18
Interfacial thickness, κ (nm)	4
E (GPa) (Young’s modulus)	202
υ (Poisson’s ratio)	0.22
